# Usefulness of BioFire FilmArray BCID2 for Blood Culture Processing in Clinical Practice

**DOI:** 10.1128/JCM.00543-21

**Published:** 2021-07-19

**Authors:** Benjamin Berinson, Anna Both, Laura Berneking, Martin Christner, Marc Lütgehetmann, Martin Aepfelbacher, Holger Rohde

**Affiliations:** a Institute for Medical Microbiology, Virology and Hygiene, University Medical Center Hamburg-Eppendorf, Hamburg, Germany; Medical College of Wisconsin

**Keywords:** sepsis, blood culture, diagnostics, multiplex PCR, FilmArray, species identification, resistance, molecular methods, rapid tests, technical evaluation

## Abstract

Rapid pathogen characterization from positive blood cultures (BC) can improve management of patients with bloodstream infections (BSI). The FilmArray blood culture identification (BCID) assay is a molecular test approved for direct identification of BSI causing pathogens from positive BC. A recently updated version of the panel (BCID2) comprises improved species identification characteristics and allows for the detection of one expanded-spectrum β-lactamase (ESBL)- and several carbapenemase-encoding genes. Here, the clinical performance of the BCID2 assay for species identification in 180 positive BCs was evaluated. BCID2 results were concordant with the standard of care (SOC) in 159/180 (88.3%) BCs; 68/74 (91.9%) and 71/74 (96.0%) of all samples growing monobacterial, Gram-positive or Gram-negative pathogens, respectively, were identified, in agreement with SOC results. Nonconcordance was related to the detection of additional pathogens by the BCID2 assay (*n* = 4), discrepant species identification (*n* = 4), or failure of BCID2 to detect on-panel pathogens (*n* = 1). A number (12/31; 38.7%) of discordant results became evident in polymicrobial BC specimens. BCID2 identified the presence of *bla*_CTX-M_-carrying species in 12 BC specimens but failed to predict third-generation cephalosporin resistance in four isolates exhibiting independent cephalosporin resistance mechanisms. Carbapenem resistance related to the presence of *bla*_VIM-2_ or *bla*_Oxa-48_-like was correctly predicted in two isolates. In conclusion, the BCID2 assay is a reliable tool for rapid BC processing and species identification. Despite inclusion of common ESBL- or carbapenemase-encoding markers, the multifactorial nature of β-lactam resistance in Gram-negative organisms warrants combination of BCID2 with (rapid) phenotypic susceptibility assays.

## INTRODUCTION

Bacteremia and sepsis are devastating diseases associated with high mortality and sequelae (1). Therefore, timely administration of effective therapy is crucially important in improving a patient’s outcome (2). The effectiveness of empirical anti-infective therapies is significantly impaired by the worldwide spread of Gram-negative and Gram-positive bacteria carrying acquired resistance determinants (3). Consequently, rapid identification of causative organisms and detection of lead resistance determinants is of major importance for targeted antimicrobial therapy and optimal patient management (4). Aiming at speeding up the processing of positive blood cultures, numerous novel technologies have been introduced into daily clinical microbiological workflows (5). Species identification of isolates from positive blood cultures may be obtained by using matrix-assisted laser desorption ionization–time-of-flight (MALDI-TOF) mass spectrometry. The broad introduction into routine workflows is, however, currently hampered by the significant workload related to the need for bacterial isolation from blood culture specimens. Additional technologies for direct species identification have emerged on the market, e.g., the Accelerate Pheno System (Accelerate Diagnostics, USA) uses a combination of gel electrofiltration and fluorescence *in situ* hybridization for fully automated bacterial identification, being provided usually within 1.5 h (6). Available molecular assays marketed for direct species identification usually provide information on some resistance markers (e.g., *mecA*, *vanA-vanB*, and expanded-spectrum β-lactamase [ESBL]- and carbapenemase-encoding genes) and enable short turnaround and hands-on times (7, 8). The BioFire FilmArray blood culture ID (BCID) assay, a highly multiplexed, single-pouch PCR kit that identifies 24 pathogens and gives additional insight into some important resistance genes (*mecA*, *vanA-vanB*, and *bla*_KPC_), proved to be a powerful tool (9, 10).

The recently released BCID2, an update to the original BCID, identifies 33 species, including a new distinction between Enterococcus faecalis and E. faecium. Furthermore, it analyzes 10 genetic resistance markers, including common carbapenemase-encoding genes (i.e., *bla*_KPC_, *bla*_IMP_, *bla*_NDM_, *bla*_OXA-48_-like, and *bla*_VIM_), the most common ESBL gene (*bla*_CTX-M_) (11), and a genetic marker for colistin resistance (*mcr-1*). Here, we evaluate the BCID2 real-life performance in a tertiary care hospital in Germany by comparison of identification results of 180 blood cultures from patients with a bloodstream infection (BSI) against our current culture-based standard of care (SOC) and an identification employing MALDI-TOF mass spectrometry.

## MATERIALS AND METHODS

### Study setting and inclusion criteria.

This prospective single-center study was conducted at the University Medical Center Hamburg-Eppendorf, Germany, a 1,700-bed tertiary-care university hospital. Blood cultures qualified for BCID2 analysis if drawn from patients hospitalized in the intensive care unit/emergency department, the age of the patient was ≥18 years, no positive blood cultures from the patient were available within the past 7 days from which pathogens of the same Gram staining behavior had been cultivated, and time to positivity (TTP) was <20 h. The TTP was set as an inclusion criterion, since a prestudy analysis at the study site revealed a predominance of coagulase-negative staphylococci after TTP of >20 h. Samples not meeting these inclusion criteria were excluded from the study. The study was conducted between 1 August 2019 and 31 October 2020, and specimens were included on weekdays from 8 a.m. to 6 p.m. In total, 198 BC specimens were tested, of which 183 met the previously defined inclusion criteria. Three BSIs grew an off-panel pathogen or the run was called invalid, leading to 180 BSIs that could be included in the clinical analysis.

### Blood culture diagnostics.

Blood culture bottles (Bactec Plus aerobic/anaerobic; BD, Heidelberg, Germany) were incubated in a BD Bactec Fx instrument (BD, Heidelberg, Germany) at 37°C. Bottles flagged positive were removed and a Gram stain was performed. From all blood culture bottles, one drop (40 μl) was streaked onto a MacConkey plate and a blood agar plate. If yeasts were detected by Gram stain, a Sabouraud agar plate (Oxoid, Basingstoke, UK) was added. Incubation was carried out at 37°C and ambient conditions for a maximum of 48 h. Furthermore, 40 μl of the blood culture medium was applied to a chocolate agar plate (Oxoid, Basingstoke, UK) and incubated for at least 4 h at 37°C in 5% CO_2_. Bacteria obtained from short cultures were subjected to pathogen identification using MALDI-TOF (Microflex; Bruker Daltonics, Bremen, Germany).

All Gram-negative organisms were also subjected to susceptibility testing on a VITEK2 instrument (bioMérieux, Marcy l´Étoil, France) using the VITEK2 AST-N223 card. ESBL phenotypes as identified by VITEK2 susceptibility testing were confirmed using the ESBL combination disk test (CDT) (cefotaxime, 30 μg; cefotaxime-clavulanic acid, 30 and 10 μg, respectively; cefoxitin, 30 μg; ceftazidime, 30 μg; ceftazidime-clavulanic acid, 30 and 10 μg, respectively; all from BD, Heidelberg, Germany) (12), combined with phenotypic AmpC detection (MAST Group, Bootle, UK), if necessary. Isolates with inconclusive phenotypic results were further characterized by an in-house PCR assay to detect *bla*_CTX-M_, *bla*_TEM_, and *bla*_SHV_ (13). Isolates exhibiting a carbapenem-resistant phenotype were tested for *bla*_OXA-48_-like, *bla*_VIM-2_, *bla*_KPC_, *bla*_NDM-1_, *bla*_IMP_, *bla*_BIC_, *bla*_GES_, *bla*_NMC-A/IMI_, and *bla*_SME_ using an in-house PCR panel (14, 15).

Specimens growing Gram-positive cocci were inoculated into a GeneXpert SA/methicillin-resistant Staphylococcus aureus (MRSA) cartridge (cluster forming cocci) or into a *vanA-vanB* cartridge (Gram stain suggestive of enterococci) (both from Cepheid, Sunnydale, CA, USA) (7). Phenotypic susceptibility of all Gram-positive isolates was tested using a VITEK2 system and the Vitek AST-P611 card (staphylococci, enterococci) or agar diffusion (streptococci) according to EUCAST protocols. Oxacillin resistance in Staphylococcus aureus was confirmed using an immunochromatographic assay (Abbot, Scarborough, ME, USA). Phenotypic glycopeptide resistance was validated using an in-house *vanA-vanB* PCR assay (16).

### FilmArray BCID2 testing.

FilmArray BCID2 testing was performed according to the manufacturer’s guidelines. Briefly, a hydration solution was loaded into the pouch, and 4 to 5 drops of the positive blood culture solution were mixed with the provided sample buffer. This mixture was applied to the pouch. This was subsequently loaded into the instrument. A nucleic acid extraction, a multiplexed nested PCR, and a product melt temperature analysis were performed by the instrument. A result could be expected within about 60 min. An overview of genera or families of bacteria and resistance markers identified by the BCID2 panel can be found online (https://www.biomerieux-diagnostics.com/biofire-bcid-panel).

### Spiking of blood culture bottles.

Strains, which were stored at −80°C, harboring two or more resistance genes were selected from our strain collection. These strains were thawed onto an agar plate. The blood culture bottles were spiked by strictly following the EUCAST RAST QC protocol (17). In brief, bacteria were suspended in 0.9% NaCl to a final turbidity equivalent of a 0.5 McFarland standard. One milliliter of a 1:1,000,000 diluted bacterial solution was then mixed with 5 ml of sterile sheep blood and inoculated into a BD Bactec Plus aerobic bottle (BD, Heidelberg, Germany). Bottles were incubated in a BD Bactec Fx instrument (BD, Heidelberg, Germany) at 37°C. After being flagged positive, the specimen underwent analysis using the BCID2 assay as described above.

### QC.

The MALDI-TOF quality control (QC) was performed on a daily basis with Escherichia coli ATCC 25922. The EUCAST quality control procedure was performed regularly once per week to control the performance of the diffusion disks and the agar used, with E. coli ATCC 25922, Klebsiella pneumoniae ATCC 700603, and P. aeruginosa ATCC 27853 (18). Furthermore, the VITEK2 QC was performed for the AST-N223 card with E. coli ATCC 25922, E. coli ATCC 35218, and K. pneumoniae ATCC 700603. For the AST-P611 card, the QC was performed with E. faecalis ATCC 29212, E. faecalis 51299, S. aureus 29213, S. aureus ATCC BAA-1026, S. aureus ATCC BAA-976, and S. aureus ATCC BAA-977. The QC for ESBL CDT was performed with K. pneumoniae ATCC 700603.

Additionally, the BCID2 QC was performed once with the supplied QC test vials.

### Ethics.

According to the Ethics Committee of the Hamburg Chamber of Physicians, no informed consent was required for the collection, analysis, and publication of these data.

## RESULTS

**FIG 1:**
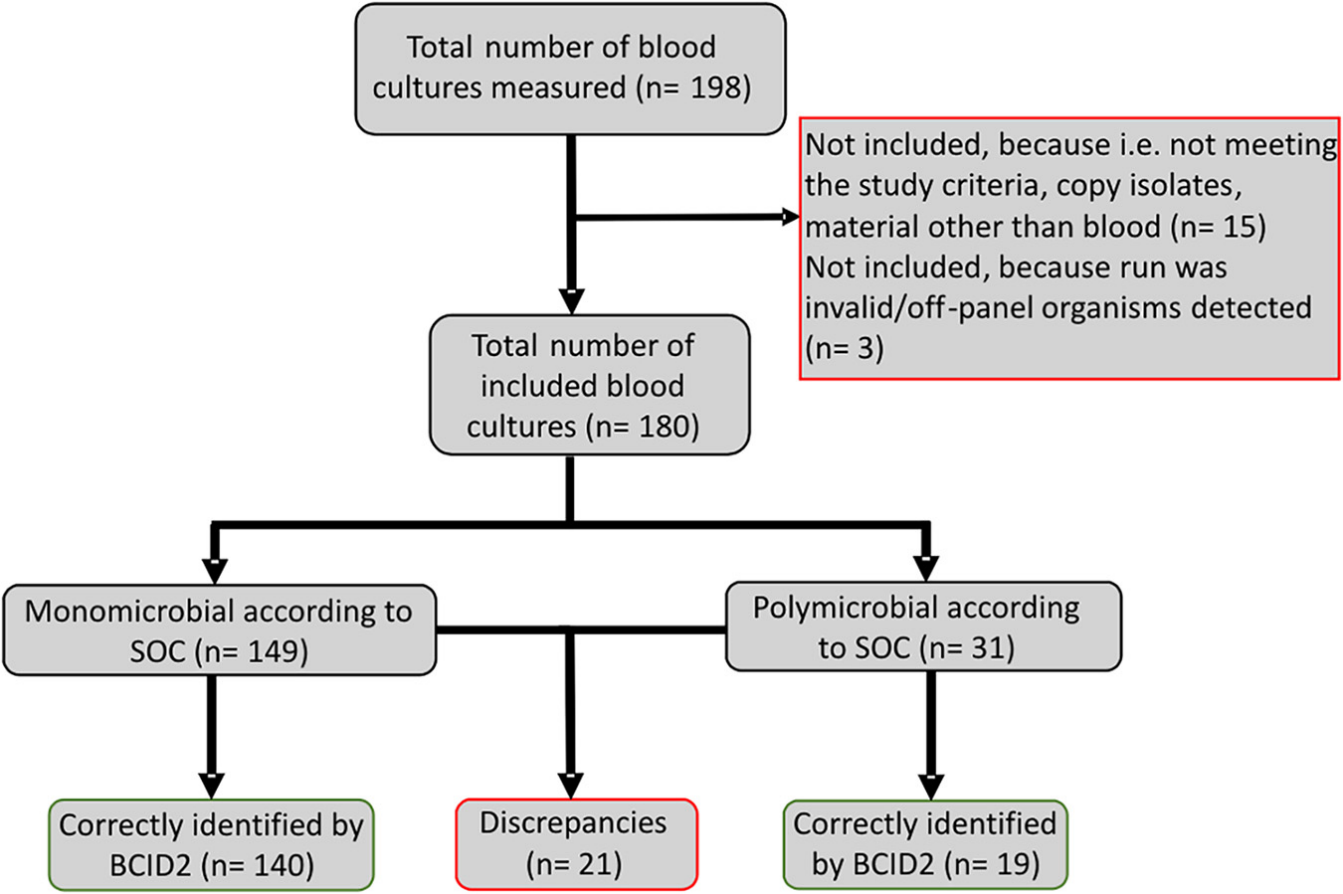
Flowchart showing the inclusion and results/interpretation of isolates. The discrepancies are presented in more detail in Table 1.

Between 1 August 2019 and 31 October 2020, 198 BCID2 tests were prospectively performed, of which 180 were included in this study. Fifteen samples had to be excluded, because they did not meet the predefined inclusion criteria or because nonblood material (e.g., pleura fluid or ascites) had been inoculated into a blood culture flask (Fig. 1). Furthermore, two monomicrobial runs revealed an off-panel organism (Enterococcus hirae and Acinetobacter beijerinckii) and one monomicrobial run was called invalid. Therefore, these have not been included in the performance analysis. The average TTP of the 180 included runs was 13.6 h (standard deviation, 3.8 h; range, 3.6 h to 20.0 h; interquartile range, 11.0 to 17.1 h; median, 13.1 h). A per-run result overview is available in Table S1 in the supplemental material. SOC detected 152 isolates in monomicrobial specimens, of which 150 were BCID2 on the panel (coverage rate, 98.7%). In 31 polymicrobial specimens, a total of 65 isolates were detected by SOC, of which 62 were BCID2 on the panel (coverage rate, 95.4%).

### Species identification.

SOC analytics revealed growth of single-species cultures in 149 of all 180 positive blood cultures (82.8%) (Fig. 1). In 74 (49.7%) Gram-positive organisms were identified in 74 (49.7%) Gram-negative organisms, and one (0.7%) blood culture grew yeast (Table S1). Of the 180 BSIs growing on-panel organisms, 159 (88.3%) samples were correctly identified by BCID2. Table 1 provides an overview of discrepant findings between SOC diagnostics and BCID2.

**TABLE 1:** Overview on discordant species identification by SOC analytics and the BCID2 assay system

Study no.	SOC identification	BCID2 identification
Monomicrobial Gram positive		
6	E. faecalis	E. faecalis, Staphylococcus spp.
47	*S. haemolyticus*	S. epidermidis
54	E. faecalis	E. faecalis, S. epidermidis
62	*S. haemolyticus*	S. epidermidis
97	*S. haemolyticus*	S. epidermidis
118	*S. haemolyticus*	S. epidermidis
Monomicrobial Gram negative		
17	K. pneumoniae	None
28	E. coli	E. coli, S. epidermidis
70	E. coli	E. coli, S. epidermidis
Polymicrobial culture		
5	K. pneumoniae, *S. capitis*	K. pneumoniae group
14	P. aeruginosa, S. maltophilia	P. aeruginosa
20	E. faecium, *S. haemolyticus*	E. faecium, S. epidermidis
51	E. faecium, S. epidermidis	E. faecium
58	E. coli, *A. veronii*	E. coli, K. pneumoniae group
73	E. coli, S. epidermidis	E. coli, Staphylococcus spp.
75	*S. haemolyticus*, C. krusei	S. epidermidis, C. krusei
82	E. coli, *S. anginosus* group	E. coli, B. fragilis, Streptococcus spp.
123	C. perfringens, S. epidermidis	None
127	E. faecalis, E. faecium, Candida albicans	E. faecalis, E. faecium
129	K. oxytoca, E. faecium	K. oxytoca
178	*P. agglomerans*, *S. haemolyticus*	*Enterobacterales*, S. epidermidis

A total of 68/74 (91.9%) of the Gram-positive monomicrobial cultures were correctly identified by the BCID2 assay. In two cases, the BCID2 identified additional coagulase-negative staphylococci that were not detected by SOC. In four cases, Staphylococcus epidermidis was identified by the BCID2 system, while SOC identified *S. haemolyticus*.

Of 74 on-panel monomicrobial Gram-negative cultures, BCID2 identified 71 (95.9%) in accordance with results from SOC. In 2/74 specimens, the BCID2 system identified additional coagulase-negative staphylococci that were not identified by SOC. No organism was identified in one specimen in which SOC diagnostics identified K. pneumoniae.

One yeast was correctly identified as Candida krusei (100%).

Of 31 polymicrobial blood cultures identified by SOC, the BCID2 analysis produced concordant results in 19 cases (61.3%). Discrepancies resulted from identification of coagulase-negative staphylococci but nonconcordant species identification (i.e., BCID2 called Staphylococcus epidermidis while SOC identified a different coagulase-negative staphylococcus species; *n* = 4), additional growth of coagulase-negative staphylococci that remained undetected by the BCID2 assay (*n* = 3), and growth of additional on-panel pathogens (*n* = 3). Furthermore, in one case BCID2 identified an additional species (Bacteroides fragilis) not detected by SOC. In one blood culture, BCID2 identified E. coli and K. pneumoniae group and the presence of *bla*_CTX-M_, while SOC found E. coli and Aeromonas veronii, both appearing phenotypically ESBL negative (study number 58) (see Table S1 in the supplemental material). Once the BCID2 identified only S. epidermidis, whereas SOC grew the S. epidermidis and Bacillus cereus group. Due to the fact that the latter represents an off-panel organism, this was not counted as a discordant result.

### Detection of resistance markers.

BCID2 can detect a variety of genetic markers associated with acquired resistance phenotypes in Gram-positive and -negative organisms. SOC identified 16 Gram-negative third-generation cephalosporin-resistant isolates: E. coli (3GCREC, *n* = 12), *K. pneumonia* group (3GCRKP, *n* = 3), K. oxytoca (3GCRKO, *n* = 1), and two isolates with a carbapenem-resistant phenotype (K. pneumoniae group, *n* = 1; P. aeruginosa, *n* = 1) (Table 2). In 12/16 (75.0%) of the 3GCR isolates, BCID2 identified *bla*_CTX-M_, allowing for correct prediction of cephalosporin-resistant phenotypes. Four out of 16 samples containing 3GCREC, 3GCRKO, or 3GCRKP isolates were *bla*_CTX-M_ negative by BCID2; thus, the system failed to correctly predict the phenotype. SOC analysis revealed that the respective isolates carried *bla*_TEM_ (E. coli, *n* = 1) and *bla*_SHV_ (K. pneumoniae, *n* = 1). One isolate carried *bla*_SHV_ in addition to *bla*_TEM_ (K. pneumoniae, *n* = 1), explaining the cephalosporin-resistant phenotype. In one isolate (K. oxytoca, *n* = 1), the mechanisms underlying the third-generation cephalosporin resistance remained unresolved. BCID2 identified *bla*_CTX-M_ in one polymicrobial blood culture that ultimately did not grow a cephalosporin-resistant species (E. coli and K. pneumoniae with detection of *bla_CTX-M_*, study number 58) (Table S1).

**TABLE 2:** Distribution of resistance markers detected by BCID2

Isolate	Resistance marker detected by BCID2 (*n*)			
	*bla*_CTX-M_	*bla*_OXA-48_-like	*bla*_VIM_	None detected
Phenotypic third-generation cephalosporin resistance				
E. coli (*n* = 12)	11	0	0	1^*a*^
K. pneumoniae group (*n* = 3)	1	0	0	2^*b*^
K. oxytoca (*n*= 1)	0	0	0	1^*c*^
Carbapenem-resistant isolates				
P. aeruginosa (*n* = 1)	0	0	1	0
K. pneumoniae group (*n* = 1)	1	1	0	0

a Molecular analysis revealed the presence of *bla*_TEM_.

b Molecular analysis revealed the presence of *bla*_SHV_ or a combination of *bla*_SHV_ and *bla*_TEM_.

c Molecular analysis did not reveal the presence of a *bla*_TEM_, *bla*_SHV_, or *bla*_CTX-M_.

During the study period, two phenotypically carbapenem-resistant Gram-negative organisms were identified, of which the correct phenotype was predicted by BCID2 identifying once *bla*_VIM_ and once *bla*_OXA-48_-like in addition to *bla*_CTX-M_ from bottles growing P. aeruginosa and K. pneumoniae, respectively.

In 15 blood cultures, methicillin-resistant Staphylococcus spp. were correctly identified by BCID2. Of note, no MRSA bacteremia was detected in our study period. Furthermore, nine VRE were detected by SOC, of which eight (88.9%) were correctly identified by BCID2. The discrepancy was related to a nondetection of E. faecium in a polymicrobial setting (Table 1).

### Spiked blood culture bottles.

The overall prevalence of ESBL- or carbapenemase-producing isolates at the study site made it impossible to draw valid conclusions on the performance of BCID2 to detect underlying genetic markers. Therefore, a series of experiments were carried out in which 10 clinical isolates with genetically characterized resistance profiles were used to spike blood cultures. These isolates included Serratia marcescens (*n* = 1), Enterobacter cloacae complex (*n* = 3), E. coli (*n* = 5), and K. oxytoca (*n* = 1) harboring at least two resistance genes, which included bla_CTX-M_, *bla*_TEM_, *bla*_OXA-48_-like, *bla*_VIM_, *bla*_NDM_, and *mcr1*.

The BCID2 identified the species and the on-panel resistance markers in all 10 isolates tested. The performance of the BCID2 is presented in Table 3.

**TABLE 3:** Usefulness of BCID2 to detect *mcr1*, *bla*_CTX-M_, or carbapenemase-encoding genes from isolates grown in blood culture bottles

Isolate (resistance determinant)	BCID2 result
S. marcescens (*bla*_OXA-48_-like, *bla*_VIM-2_, *bla_CTX-M_*)^*a*^	S. marcescens (*bla*_OXA-48_-like, *bla*_VIM_, *bla*_CTX-M_)
E. cloacae complex (*bla*_OXA-48_-like, *bla*_NDM_)^*a*^	E. cloacae complex (*bla*_OXA-48_-like, *bla*_NDM_)
E. cloacae complex (*bla*_CTX-M-3_, *bla*_OXA-48_, *bla*_TEM-1_)^*b*^	E. cloacae complex (*bla*_CTX-M_, *bla*_OXA-48_-like)
E. cloacae complex (*bla*_CTX-M-9_, *bla*_OXA-48_)^*b*^	E. cloacae complex (*bla*_CTX-M_, *bla*_OXA-48_-like)
E. coli (*bla*_CTX-M-15_, *bla*_OXA-244_)^*b*^	E. coli (*bla*_CTX-M_, *bla*_OXA-48_-like)
E. coli (*bla*_CTX-M-24_, *bla*_OXA-48_)^*b*^	E. coli (*bla*_CTX-M_, *bla*a_OXA-48_-like)
E. coli (*bla*_NDM-5_, *bla*_TEM-1_)^*b*^	E. coli (*bla*_NDM_)
E. coli (*bla*_CTX-M-3_, *bla*_OXA-181_, *bla*_TEM-35_)^*b*^	E. coli (*bla*_CTX-M_, *bla*_OXA-48_-like)
E. coli (*bla*_CTX-M-1_, *mcr1.1*)^*b*^	E. coli (*bla*_CTX-M_, *mcr1*)
K. oxytoca (*bla*_CTX-M-15_, *bla*_VIM-4_)^*b*^	K. oxytoca (*bla*_CTX-M_, *bla*a_VIM_)

a β-Lactamase-encoding genes were detected by PCR (15).

b β-Lactamase-encoding genes were identified from whole-genome assemblies using abricate (https://github.com/tseemann/abricate) and the NCBI bacterial antimicrobial resistance reference gene database (https://www.ncbi.nlm.nih.gov/bioproject/313047).

## DISCUSSION

The rapid identification of the causative pathogen in BSI and its possible resistance markers is crucial for timely initiation of effective antibiotic therapy. This can improve patient outcome, reduce mortality, and spare usage of broad-spectrum antibiotics (10, 19–21). The BCID2 showed good overall concordance with conventional species identification using MALDI-TOF mass spectrometry, particularly in monomicrobial cultures (94.0% correctly identified organisms). Most of the observed discordant results were due to wrongly or additionally identified coagulase-negative staphylococci. Ambiguous results do not necessarily relate to technical limitations of the BCID2 assay. It needs to be taken into account that by eye, colonies of different coagulase-negative staphylococcal species are often indistinguishable; thus, it cannot be excluded that at least some discordant results can be explained by not selecting the correct colony for MALDI-TOF-based species analysis. Furthermore, faster growth of Gram-negative bacteria might have inhibited or masked the growth of additional Gram-positive organisms that were then only detected by molecular BCID2 analysis. Of notice, an urgent field safety notice was released by bioMérieux due to detection of Proteus species DNA in Proteus species-negative blood culture bottles in February 2020 (22). This highlights an important issue in molecular assays, since nonviable organisms or DNA could lead to false-positive results.

Limitations of the BCID2 assay system became apparent during analysis of polymicrobial cultures, a finding that, to various extents, was previously reported for the BCID1 assay (23–25). Compared to results from cultural analysis, discordant BCID2 results were related to misidentified species or the additional identification of bacterial species by conventional growth on agar plates. Data on the performance of BCID2 in a polymicrobial setting are sparse. One study retrospectively analyzed stored frozen blood culture specimens, also including polymicrobial samples. The study showed a 100% species identification rate (26).

A significant improvement of the BCID2 panel compared to BCID1 is the built-in ability to differentiate between E. faecalis and E. faecium. In combination with the ability to detect *vanA-vanB*, this property could prove useful in antibiotic stewardship program (ASP) interventions, allowing to shorten empirical vancomycin therapies in E. faecalis infections or begin early escalation in VRE BSI.

A potential strength of the BCID2 assay and importance for ASP arises from the ability to detect key genetic markers underlying cephalosporin and carbapenem resistance in Gram-negative organisms. In fact, the most common determinants of third-generation cephalosporin and carbapenem resistance in E. coli and K. pneumoniae are covered by the assay. However, given the multifactorial molecular basis of cephalosporin and carbapenem resistance in Gram-negative species, limitations of genetic assays in predicting susceptibilities against β-lactams are evident. Here, this was exemplified by the failure of BCID2 to correctly predict CTX-M-independent cephalosporin resistance in four 3GCREC, 3GCRKO, and 3GCRKP isolates. Importantly, carbapenem resistance in Enterobacter spp. and P. aeruginosa is related to carbapenemase-independent mechanisms in around 80% of isolates (15). Although BCID2 correctly identified carbapenemases in two clinical specimens and blood cultures spiked with carbapenemase-producing *Enterobacterales*, it is reasonable to speculate that broader routine use of BCID2 will result in relevant error rates in the valid prediction of carbapenem susceptibilities in Gram-negative isolates. Therefore, at present, available rapid phenotypic susceptibility testing assay formats (e.g., Accelerate and EUCAST RAST) offer a broader and, thus, more reliable approach toward early reporting of resistance phenotypes (27, 28).

Our study is limited by its monocentric design and limited number of isolates. In particular, rare isolates like Listeria monocytogenes and Neisseria meningitidis were not detected in our study. Given the low prevalence of MRSA, 3GCR, and carbapenem-resistant isolates causing BSIs at the study site (rate in invasive isolates in 2018, 10.9% for MRSA, 18.4% and 13.0% for 3GCREC and 3GCRKP, respectively, and 0.6% and 0.01% for carbapenem-resistant E. coli and K. pneumoniae, respectively), only a limited number of specimens containing organisms with on-panel molecular determinants associated with resistance phenotypes were included. To get an impression of the ability of the BCID2 to detect beta-lactamases and other resistance genes, blood cultures were spiked with isolates carrying defined resistance markers. MRSA was not used for spiking experiments, given available data on MRSA detection by the BCID (9). Another limitation is that discrepant results were not reanalyzed due to the unavailability of leftover blood culture material for follow-up analysis. Furthermore, our study did not include pediatric blood cultures. Further studies in a multicenter setting are warranted to determine the usefulness and possible problems of the BCID2, especially concerning its effect on patient management and outcomes. In fact, a recent study showed, in a retrospective analysis, a superiority of BCID2 to BCID1 and Verigene BC-Gram negative for theoretical optimal antimicrobial prescribing decisions (29).

In conclusion, BCID2 proves to be a reliable assay for rapid identification of BSI-causing organisms from positive blood cultures. The promise of direct antimicrobial resistance phenotype prediction in Gram-negative microorganisms is inherently limited by their multifactorial functional basis, which is currently not accessible by PCR assays. Here, complementation with rapid phenotypic assay formats is necessary.
