# Supplementary material for: Usefulness of BioFire FilmArray BCID2 for Blood Culture Processing in Clinical Practice
Source: J Clin Microbiol. 2021 Jul 19;59(8):e00543-21. doi: 10.1128/JCM.00543-21 (PMC8373244; doi:10.1128/JCM.00543-21)
Supplement: Supplemental file 1 — Table S1. Download JCM.00543-21-s0001.pdf, PDF file, 159 KB [file jcm.00543-21-s0001.pdf]

Supplementary material

Table S1: Comparison of species ID and if applicable resistance patterns/genes following SOC work-flows and BCID2

Study number	ID Maldi (+ if applicable resistance type/gene)	ID BCID2 (resistance determinant; if applicable)
1	*Streptococcus anginosus Group*	*Streptococcus spp.*
2	*Streptococcus agalactiae*	*Streptococcus agalactiae*
3	*Escherichia coli*	*Escherichia coli*
4	*Klebsiella pneumoniae*	*Klebsiella pneumoniae Group*
5	*Klebsiella pneumoniae, Staphylococcus capitis*	*Klebsiella pneumoniae group*
6	*Enterococcus faecalis*	*Enterococcus faecalis, Staphylococcus spp.*
7	*Providencia rettgeri, Staphylococcus epidermidis* (*mecA/C*), *Stretptococcus agalactiae*	*Enteric bacteria, Staphylococcus epidermidis (mecA/C), Stretptococcus agalactiae*
8	*Escherichia coli*	*Escherichia coli*
9	*Escherichia coli*	*Escherichia coli*
10	*Escherichia coli*	*Escherichia coli*
11	*Enterococcus faecium*	*Enterococcus faecium*
12	*Enterococcus faecalis, Staphylococcus aureus*	*Enterococcus faecalis, Staphylococcus aureus*
13	*Escherichia coli*	*Escherichia coli*
14	*Pseudomonas aeruginosa* ($bla_{\text{VIM-2}}$), *Stenotrophomas maltophilia*	*Pseudomonas aeruginosa* (bla_{VIM})
15	*Escherichia coli* (3GCR)	*Escherichia coli* ($bla_{CTX\text{-}M}$)

16	*Streptococcus pyogenes*	*Streptococcus pyogenes*
17	*Klebsiella pneumoniae*	none
18	*Streptococcus salivarius Group*	*Streptococcus spp.*
19	*Serratia marcescens*	*Serratia marcescens*
20	*Enterococcus faecium, Staphylococcus haemolyticus* (mecA/C)	*Enterococcus faecium, Staphylococcus epidermidis* (mecA/C)
21	*Pseudomonas aeruginosa*	*Pseudomonas aeruginosa*
22	*Streptococcus pneumoniae*	*Streptococcus pneumoniae*
23	*Escherichia coli*	*Escherichia coli*
24	*Escherichia coli*	*Escherichia coli*
25	*Enterococcus faecalis*	*Enterococcus faecalis*
26	*Enterococcus faecium*	*Enterococcus faecium*
27	*Haemophilus influenzae, Staphylococcus epidermidis* (*mecA/C*)	*Haemophilus influenzae, Staphylococcus epidermidis* (*mecA/C*)
28	*Escherichia coli*	*Escherichia coli, Staphylococcus epidermidis* (*mecA/C*)
29	*Streptococcus salivarius Group*	*Streptococcus spp.*
30	*Escherichia coli* (3GCR)	*Escherichia coli* (*bla*$_{CTX\text{-}M}$)
31	*Klebsiella pneumoniae* (*bla*$_{oxa\text{-}48}$)	*Klebsiella pneumoniae group* (*bla*$_{CTX\text{-}M}$, (*bla*$_{oxa\text{-}48}$-like
32	*Candida krusei*	*Candida krusei*

33	*Enterobacter cloacae* Complex	*Enterobacter cloacae* Complex
34	*Staphylococcus hominis*	*Staphylococcus spp.*
35	*Escherichia coli*	*Escherichia coli*
36	*Streptococcus mitis Group, Staphylococcus hominis*	*Staphylococcus spp. Streptococcus spp.*
37	*Streptococcus pneumoniae*	*Streptococcus pneumoniae*
38	*Staphylococcus aureus*	*Staphylococcus aureus*
39	*Escherichia coli* (3GCR)	*Escherichia coli*
40	*Enterobacter cloacae* Complex	*Enterobacter cloacae* Complex
41	*Klebsiella pneumoniae*	*Klebsiella pneumoniae Group*
42	*Enterococcus faecium*	*Enterococcus faecium*
43	*Escherichia coli*	*Escherichia coli*
44	*Escherichia coli*	*Escherichia coli*
45	*Enterococcus faecium (vanB)*	*Enterococcus faecium (vanA/B)*
46	*Staphylococcus aureus*	*Staphylococcus aureus*
47	*Staphylococcus haemolyticus* (*mecA/C*)	*Staphylococcus epidermidis* (*mecA/C*)
48	*Pseudomonas aeruginosa*	*Pseudomonas aeruginosa*
49	*Escherichia coli, Enterococcus faecium*	*Escherichia coli, Enterococcus faecium*

50	*Streptococcus dysgalactiae*	*Streptococcus spp.*
51	*Enterococcus faecium, Staphylococcus epidermidis*	*Enterococcus faecium*
52	*Streptococcus dysgalactiae*	*Streptococcus spp.*
53	*Enterococcus faecium (vanB), Staphylococcus haemolyticus*	*Enterococcus faecium (vanA/B), Staphylococcus spp.*
54	*Enterococcus faecalis*	*Enterococcus faecalis, Staphylococcus epidermidis* (*mecA/C*)
55	*Enterococcus faecium (vanB)*	*Enterococcus faecium (vanA/B)*
56	*Escherichia coli* (3GCR)	*Escherichia coli* ($bla_{CTX\text{-}M}$)
57	*Escherichia coli*	*Escherichia coli*
58	*Escherichia coli, Aeromonas veronii*	*Escherichia coli, Klebsiella pneumoniae Group,* ($bla_{CTX\text{-}M}$)
59	*Enterobacter cloacae* Complex	*Enterobacter cloacae* Complex
60	*Proteus mirabilis*	*Proteus spp.*
61	*Staphylococcus aureus*	*Staphylococcus aureus*
62	*Staphylococcus haemolyticus* (*mecA/C*)	*Staphylococcus epidermidis* (*mecA/C*)
63	*Streptococcus sanguinis Group*	*Streptococcus spp.*
64	*Escherichia coli, Staphylococcus epidermidis* (*mecA/C*)	*Escherichia coli, Staphylococcus epidermidis* (*mecA/C*)
65	*Streptococcus agalactiae*	*Streptococcus agalactiae*
66	*Escherichia coli*	*Escherichia coli*

67	*Escherichia coli*	*Escherichia coli*
68	*Staphylococcus aureus*	*Staphylococcus aureus*
69	*Streptococcus pneumoniae*	*Streptococcus pneumoniae*
70	*Escherichia coli* (3GCR)	*Escherichia coli* (*bla*$_{CTX-M}$), *Staphylococcus epidermidis* (mecA/C)
71	*Staphylococcus aureus*	*Staphylococcus aureus*
72	*Escherichia coli*	*Escherichia coli*
73	*Escherichia coli, Staphylococcus epidermidis*	*Escherichia coli, Staphylococcus spp.*
74	*Enterococcus faecium (vanB)*	*Enterococcus faecium (vanA/B)*
75	*Staphylococcus haemolyticus (mecA/C), Candida krusei*	*Staphylococcus epidermidis (mecA/C), Candida krusei*
76	*Enterococcus faecalis, Staphylococcus epidermidis*	*Enterococcus faecalis, Staphylococcus epidermidis*
77	*Staphylococcus haemolyticus*	*Staphylococcus spp.*
78	*Escherichia coli*	*Escherichia coli*
79	*Enterococcus faecium (vanB), Candida albicans, Staphylococcus epidermidis (mecA/C)*	*Enterococcus faecium (vanA/B), Staphylococcus epidermidis (mecA/C), Candida albicans*
80	*Streptococcus dysgalactiae*	*Streptococcus spp.*
81	*Enterococcus faecium (vanB)*	*Enterococcus faecium (vanA/B)*
82	*Escherichia coli* (3GCR), *Streptococcus anginosus Group*	*Escherichia coli* (*bla*$_{CTX-M}$), *Bacteroides fragilis, Streptococcus spp.*
83	*Escherichia coli*	*Escherichia coli*

84	*Staphylococcus aureus*	*Staphylococcus aureus*
85	*Streptococcus pneumoniae*	*Streptococcus pneumoniae*
86	*Staphylococcus aureus*	*Staphylococcus aureus*
87	*Streptococcus agalactiae, Staphylococcus epidermidis*	*Streptococcus agalactiae, Staphylococcus epidermidis*
88	*Streptococcus equinus/bovis* Group	*Streptococcus spp.*
89	*Escherichia coli* (3GCR)	*Escherichia coli* ($bla_{CTX\text{-}M}$)
90	*Escherichia coli* (3GCR), *Pseudomonas aeruginosa*	*Escherichia coli* ($bla_{CTX\text{-}M}$), *Pseudomonas aeruginosa*
91	*Escherichia coli*	*Escherichia coli*
92	*Escherichia coli*	*Escherichia coli*
93	*Staphylococcus aureus*	*Staphylococcus aureus*
94	*Staphylococcus aureus*	*Staphylococcus aureus*
95	*Escherichia coli*	*Escherichia coli*
96	*Staphylococcus epidermidis (mecA/C), Staphylococcus caprae*	*Staphylococcus epidermidis* (*mecA/C*)
97	*Staphylococcus haemolyticus*	*Staphylococcus epidermidis*
98	*Staphylococcus epidermidis, Bacillus cereus Group*	*Staphylococcus epidermidis*
99	*Staphylococcus haemolyticus*	*Staphylococcus spp.*
100	*Klebsiella oxytoca*	*Klebsiella oxytoca*

101	*Staphylococcus hominis, Staphylococcus epidermidis*	*Staphylococcus epidermidis*
102	*Enterococcus faecalis*	*Enterococcus faecalis*
103	*Staphylococcus aureus*	*Staphylococcus aureus*
104	*Staphylococcus hominis*	*Staphylococcus spp.*
105	*Klebsiella oxytoca*	*Klebsiella oxytoca*
106	*Enterococcus faecium*	*Enterococcus faecium*
107	*Klebsiella pneumoniae*	*Klebsiella pneumoniae Group*
108	*Enterococcus faecalis*	*Enterococcus faecalis*
109	*Enterococcus hirae*	none
110	*Acinetobacter baumannii complex*	*Acinetobacter calcoaceticus-baumannii Complex*
111	*Escherichia coli*	*Escherichia coli*
112	*Enterococcus faecium (vanB)*	*Enterococcus faecium (vanA/B)*
113	*Escherichia coli* (3GCR)	*Escherichia coli* (bla_{CTX-M})
114	*Proteus mirabilis*	*Proteus spp.*
115	*Enterococcus faecium*	*Enterococcus faecium*
116	*Klebsiella pneumoniae* (3GCR)	*Klebsiella pneumoniae Group*
117	*Escherichia coli*	*Escherichia coli*

118	*Staphylococcus haemolyticus*	*Staphylococcus epidermidis* (*mecA/C*)
119	*Staphylococcus haemolyticus*	*Staphylococcus spp.*
120	*Proteus mirabilis*	*Proteus spp.*
121	*Staphylococcus haemolyticus*	*Staphylococcus spp.*
122	*Staphylococcus hominis*	*Staphylococcus spp.*
123	*Clostridium perfringens, Staphylococcus epidermidis*	none
124	*Enterococcus faecium (vanA)*	*Enterococcus faecium (vanA/B)*
125	*Staphylococcus aureus*	*Staphylococcus aureus*
126	*Escherichia coli*	*Escherichia coli*
127	*Enterococcus faecalis, Enterococcus faecium, Candida albicans*	*Enterococcus faecalis, Enterococcus faecium*
128	*Streptococcus equinus/bovis* Group	*Streptococcus spp.*
129	*Klebsiella oxytoca, Enterococcus faecium (vanB)*	*Klebsiella oxytoca*
130	*Streptococcus pyogenes*	*Streptococcus pyogenes*
131	*Escherichia coli*	*Escherichia coli*
132	*Escherichia coli*	*Escherichia coli*
133	*Pseudomonas aeruginosa*	*Pseudomonas aeruginosa*
134	*Pseudomonas aeruginosa*	*Pseudomonas aeruginosa*

135	*Klebsiella oxytoca* (3GCR)	*Klebsiella oxytoca*
136	*Klebsiella pneumoniae*	*Klebsiella pneumoniae group*
137	*Streptococcus pneumoniae*	*Streptococcus pneumoniae*
138	*Klebsiella pneumoniae*	*Klebsiella pneumoniae group*
139	*Staphylococcus aureus*	*Staphylococcus aureus*
140	*Staphylococcus epidermidis*	*Staphylococcus epidermidis*
141	*Escherichia coli*	*Escherichia coli*
142	*Enterococcus faecium (vanB)*	invalid
143	*Streptococcus dysgalactiae*	*Streptococcus spp.*
144	*Klebsiella pneumoniae* (3GCR)	*Klebsiella pneumoniae group*
145	*Escherichia coli, Enterococcus faecium*	*Escherichia coli, Enterococcus faecium*
146	*Escherichia coli*	*Escherichia coli*
147	*Escherichia coli*	*Escherichia coli*
148	*Streptococcus dysgalactiae*	*Streptococcus spp.*
149	*Streptococcus mitis* Group	*Streptococcus spp.*
150	*Escherichia coli*	*Escherichia coli*
151	*Enterobacter cloacae* Complex	*Enterobacter cloacae* Complex

152	*Staphylococcus haemolyticus*	*Staphylococcus spp.*
153	*Escherichia coli* (3GCR)	*Escherichia coli* (*bla*$_{\text{CTX-M}}$)
154	*Enterobacter cloacae* Complex	*Enterobacter cloacae* Complex
155	*Klebsiella aerogenes*	*Klebsiella aerogenes*
156	*Morganella morganii*	Enteric bacteria
157	*Escherichia coli, Staphylococcus aureus*	*Escherichia coli, Staphylococcus aureus*
158	*Klebsiella pneumoniae* (3GCR)	*Klebsiella pneumoniae group*
159	*Staphylococcus haemolyticus*	*Staphylococcus spp.*
160	*Streptococcus dysgalactiae*	*Streptococcus spp.*
161	*Staphylococcus hominis*	*Staphylococcus spp.*
162	*Staphylococcus aureus*	*Staphylococcus aureus*
163	*Proteus mirabilis*	*Proteus spp.*
164	*Proteus mirabilis*	*Proteus spp.*
165	*Staphylococcus epidermidis* (*mecA/C*)	*Staphylococcus epidermidis* (*mecA/C*)
166	*Escherichia coli* (3GCR)	*Escherichia coli* (*bla*$_{\text{CTX-M}}$)
167	*Staphylococcus caprae*	*Staphylococcus spp.*
168	*Staphylococcus haemolyticus*	*Staphylococcus spp.*

169	*Pseudomonas aeruginosa*	*Pseudomonas aeruginosa*
170	*Staphylococcus hominis*	*Staphylococcus spp.*
171	*Klebsiella oxytoca*	*Klebsiella oxytoca*
172	*Acinetobacter beijerinckii*	none
173	*Streptococcus pneumoniae*	*Streptococcus pneumoniae*
174	*Escherichia coli*	*Escherichia coli*
175	*Proteus mirabilis, Staphylococcus aureus*	*Proteus spp., Staphylococcus aureus*
176	*Escherichia coli* (3GCR)	*Escherichia coli* ($bla_{\text{CTX-M}}$)
177	*Staphylococcus hominis*	*Staphylococcus spp.*
178	*Pantoea agglomerans, Staphylococcus haemolyticus*	Enteric bacteria*, Staphylococcus epidermidis* (*mecA/C*)
179	*Staphylococcus epidermidis (mecA/C), Staphylococcus hominis*	*Staphylococcus epidermidis* (*mecA/C*)
180	*Streptococcus dysgalactiae*	*Streptococcus spp.*
181	*Klebsiella pneumoniae, Pseudomonas aeruginosa*	*Klebsiella pneumoniae group, Pseudomonas aeruginosa*
182	*Enterococcus faecium*	*Enterococcus faecium*
183	*Klebsiella pneumoniae*	*Klebsiella pneumoniae Group*
